# Editorial: Developments in Animal Health Surveillance

**DOI:** 10.3389/fvets.2020.637364

**Published:** 2021-01-25

**Authors:** Marta Hernández-Jover, Bernard J. Phiri, Lesley Stringer, Marta Martínez Avilés

**Affiliations:** ^1^School of Animal and Veterinary Sciences and Graham Centre for Agricultural Innovation, Charles Sturt University, Wagga Wagga, NSW, Australia; ^2^Ministry for Primary Industries, Wellington, New Zealand; ^3^Independent Consultant, Isle of Cumbrae, Scotland, United Kingdom; ^4^Centro de Investigación en Sanidad Animal (CISA-INIA), Madrid, Spain

**Keywords:** epidemiology, monitoring, early detection, emerging diseases, freedom from disease, One Health

## Introduction

The emergence of new pathogens, and other threats to public and animal health, offers opportunities to develop innovative surveillance methods, particularly when implementing “One health” surveillance, an approach in which at least two sectors from human health, animal health (wildlife and domestic animals), plant health, food safety or environmental health collaborate to improve outcomes for all (1). The approach allows development of methods to drive economic sustainability of surveillance systems, and to optimize efficiency, considering resources are limited.

The broad scope and wide-ranging contributions from the animal and public health sectors in this Research Topic means readers will find animal health surveillance developments for zoonoses of ruminant, swine, poultry, companion animal, and primate origin. Altogether 145 authors contributed to 21 original research articles, one brief research report, one perspective article and two reviews, from 19 different countries of all five continents. The two reviews and two original research articles on rabies and West Nile disease describe, specifically, a “One health” approach to surveillance. Four articles focus on different aspects of syndromic surveillance, from design to analyses, interpretation and evaluation. The use of secondary data to describe risk factors and demographics for risk-based surveillance (outbreak preparedness and surveillance design) is further explored in five other articles. Six articles discuss the critical role of surveillance end-users and describe how to enhance disease reporting, engagement and surveillance output communication. Finally, five articles focus on developments in diagnostics with regards to evaluating assays, new sampling approaches, test characteristics and disease measure.

The development of new methods to support sustainable animal health surveillance systems requires accurate cost and benefit evaluation and assessment of cost-effectiveness. Although economic efficiency of surveillance is recognized as highly relevant, one of the studies (Häsler et al.) identified that there are few quantitative economic evaluations carried out in practice. This finding is supported by the lack of submissions describing economic evaluation of animal health surveillance to this Research Topic.

## Developments in One Health Surveillance

Rabies is a classical disease in which the “One health” concept is easily applied. Despite being a well-known fatal zoonosis, it is neglected in endemic countries, mostly in Africa and Asia. With a target of zero human deaths from dog-mediated rabies set by the World Health Organization (WHO), Food and Agriculture Organization (FAO), and World Organization for Animal Health (OIE) for 2030 (2), strengthening rabies surveillance and control in these countries is needed. An integrated, collaborative, “One health” approach to bite management between public health and veterinary sectors should ensure rapid testing of any animal that bites a person. Timely communication between health centers and veterinary field workers can be facilitated by mobile phone technology (Lushasi et al.). Benavides et al. used passive national surveillance data on bite patients over a 10-years period to identify areas and species with high potential risk of rabies transmission to humans across Brazil. The study reports bites by domestic dogs and cats being more prevalent than those caused by bats, primates, herbivores, and foxes, with an uneven incidence across the country and a relatively low level of adequate post-exposure prophylaxis administration.

The WHO, FAO and OIE have established that more than three quarters of the new human diseases at the beginning of the 21st Century have emerged or re-emerged from animals (3). While the origin of the current pandemic of severe acute respiratory syndrome coronavirus (SARS-CoV) infection remains to be elucidated, Devaux et al. report that most of the human pandemics to date are zoonoses, and review zoonoses that originate from non-human primates (NHP). Drivers of interspecies pathogen transmission from NHP to humans include incidents with NHP (in wildlife ecosystem or after illegal import of live NHP), contact with NHP carcasses, or NHP consumption (local consumption and illegal import of NHP meat).

Disease surveillance in wildlife populations is challenging because wild animals are not as closely monitored as domesticated. Instead, surveillance relies on voluntary, often opportunistic reporting from those in close contact with wild animals such as hunters, conservationists and enthusiastic citizens. However, the motivation to report varies among these groups and understanding the drivers behind the reporting patterns will lead to better interpretation of the surveillance data. For instance, disease clusters detected through citizen-derived data should be interpreted with human socio-demographics factors in mind (Thomas-Bachli et al.).

Hernandez et al. describe a “One health” surveillance in practice in the United States in an outbreak investigation context. *Clostridioides difficile* is regarded as a nosocomial pathogen of hospitals. However, the number and severity of *Clostridioides difficile* infections (CDI) outside the hospital environment or in individuals with onset of symptoms 48 h or less after hospital admission is increasing. This emerging public health concern in community settings has been linked to a potential household pet source, with molecular genetic screening of patients and their pets as a useful tool to inform appropriate preventive and control strategies.

## Developments in Data Use and Analysis for Animal Health Surveillance

### Syndromic Surveillance

The robustness of any animal health surveillance system is enhanced by the variety of its data sources and number of components. Hence, the use of data recorded for other purposes to create additional components such as syndromic surveillance is growing. Syndromic surveillance systems are real-time systems that can contribute to enhance traditional passive surveillance systems. Syndromic surveillance aims to identify aberrations in health-related events in a population that highlight unexpected trends of endemic diseases or signal the presence of an emerging disease.

There are practical challenges in transforming data into a format appropriate for syndromic surveillance and in developing suitable analyses for aberration detection. The process often requires incremental steps in an iterative cycle. Tongue et al. investigate the use of ovine fallen stock records to detect temporal and spatial aberrations in sheep mortality in the UK. Sala et al. evaluate the use of a syndromic surveillance system that incorporates a “severity” alarm to interpret excess mortality events using cattle fallen stock records. They identified important issues for optimal functionality, such as avoiding delays in notifications to the system, the need for sufficient human, financial, and legal resources to sustain it, and the need for high coverage to obtain meaningful results. Nonetheless, the system was useful to monitor seasonal diseases, quantify absence of disease or identify atypical excess mortality events. Veldhuis et al. explore routinely collected bulk milk records to identify potential aberrations. A variety of statistical methods can be used for this purpose, but choosing the appropriate method is not trivial as they vary broadly in performance and complexity. Selection of syndromes to monitor and frequency of data collection are also important in maximizing timeliness and sensitivity of aberration detection. Faverjon et al. estimate the potential value of different time series methods for building a national syndromic surveillance system for cattle in Switzerland. They suggest that detection performance is dependent on the characteristics of the syndrome time series, the nature of the epidemic and the event detection algorithm and recommend that syndrome time series be assessed through optimal detection algorithms and detection performance evaluated, before being included in an early detection surveillance system.

### Risk-Based Analytical Techniques

An in-depth understanding of animal demographics, social network structure and potential disease transmission pathways can help improve surveillance design and outbreak preparedness. By identifying populations, areas and time in which early detection of a disease outbreak is most likely to be achieved, resources for animal disease surveillance can be appropriately deployed to yield maximum benefits. This is particularly important in countries with limited resources.

As part of disease outbreak response preparedness, social network analysis can reveal influential nodes to be targeted in limiting disease spread quickly and efficiently. This is essential for rapidly spreading diseases that impact international trade such as foot and mouth disease and African swine fever (O'Hara et al.). Equally important is to identify the most crucial strategy to prevent an epidemic. Di Pillo et al. applied a stochastic model of disease transmission and a combination of intervention strategies to find that enhancing passive surveillance in owners of backyard poultry production systems was the most crucial prevention strategy to prevent highly pathogenic avian influenza epidemics in a high-density poultry area.

The use of risk-based analysis to enhance detection is described by Tzy-yun Teng et al. through a Bayesian spatial model that considers antimicrobial resistance surveillance data on *Salmonella* in pigs together with pig farm distribution, size and management. Authors identify areas at higher risk of infection and the distribution of specific serotypes, both of which can optimize future sampling. The article by Alba et al. evaluates a real time reporting system that aims to optimize clinical diagnosis in highly industrialized swine farms by incorporating information on trends and summaries of clinical events within a geographical area, together with other important demographic information characterizing the subpopulations affected. Veterinarians gain considerable advantages by using this system, but presumptive diagnoses should be confirmed with laboratory test results particularly to discard suspicions of diseases notifiable to the OIE.

Risk-based analysis can also be used to improve the interpretation of surveillance outputs at fixed locations such slaughterhouses. Stirling et al. analyzed movement data to identify and characterize potential sources of bias in slaughterhouse-based surveillance.

## Developments in Reporting and Communication of Animal Health Surveillance

### Reporting

Surveillance is intended to produce outputs to be acted upon by decision makers and other stakeholders. Data and knowledge sharing is a key element supporting surveillance programs for disease control and eradication. For surveillance to be relevant and encourage wider disease reporting, it is important to understand the needs of its stakeholders and knowledge gaps that may exist. For example, livestock producers living in remote areas should be reached and engaged in surveillance. Again, this is particularly important in resource-deprived countries where the main surveillance stakeholders are farmers who may have limited understanding of disease risk factors and transmission.

In these circumstances, a participatory investigation could be used to gather data, improve animal disease knowledge and enhance surveillance sensitivity (Ghafar et al.). An Ethiopian participatory study (Alemu et al.) investigated small ruminant disease priorities from a smallholder perspective, aiming to inform interventions to address disease-related production loss and its impact on communities. A mixed-methods approach identified respiratory diseases, gastrointestinal parasites and neurological diseases of livestock as priorities, while gaining an understanding of why the diseases are considered important to livestock owners and the extent of their understanding of disease control. The work highlighted contrasting priorities at national and community level and the large potential positive impact of production disease interventions on smallholder households. Smallholder livestock producers exist in every country in the world and are a heterogeneous group of producers. In Hernandez-Jover et al.'s cross-sectional study followed by group interviews, the authors provide an insight into animal health management practices. A delay in reporting to veterinarians could occur because most smallholder livestock producers attempt to treat symptoms by themselves or are only concerned with endemic diseases. Importantly, animal welfare was found to be a driving motivation for disease prevention, as well as sharing experiences and information between the producers.

Finally, Perez et al. present a unique example of a self-organized voluntary data sharing system to inform disease surveillance led by the livestock industry to generate scientific-driven solutions for emerging swine health issues in North America.

### Communication

Coordinated surveillance policies generally include harmonized approaches that makes the interpretation and comparison of outcomes possible. To further improve the detail, transparency, consistency and open access of surveillance reporting, Comin et al. produced a wiki that includes a provisional checklist of items that could be expanded at any time to accommodate realities different to the European context. Researchers from the European SANTERO project assessed the practical adoption of surveillance standards in Europe, and explored how to ensure innovative research reaches, and is adopted by, surveillance practitioners (Häsler et al.). They found a multiplicity of channels used to source information, and considerable heterogeneity in the adoption of recommended surveillance standards and innovative approaches among European Union, European Economic Area and Schengen countries. Although economic efficiency was considered highly relevant, few quantitative economic evaluations are carried out in practice, constraints including a skills deficit and limited resources. Recommendations included a collaborative international exchange platform for surveillance knowledge, design and dissemination of standards.

## Developments in Laboratory Detection of Animal Health Problems

Hazard-specific surveillance requires the correct laboratory identification of pathogens to tailor specific interventions to reduce or prevent their occurrence. It is therefore fundamental to analyse diagnostic test performance and evaluate new sampling strategies to reduce costs or increase population coverage.

Baruch et al. contributed evidence to inform a new approach to bovine brucellosis surveillance in Uruguay, using pooled-sera sampling. They estimated the analytic sensitivity of an indirect ELISA test for *B. abortus* for different pool sizes demonstrating that, in principle, the method could be applied to low-risk bovine populations.

Bovine tuberculosis (bTB), caused by *Mycobacterium bovis*, is endemic in most developing countries, whereas it has been eradicated from many developed countries. However, success of eradication programs differs globally, with limitations of available diagnostic tests being one of the factors impacting this success. Barandiaran et al. assessed the accuracy of a PCR-based rapid diagnostic test and compared it with that of bacteriological culture for bTB in pigs using a Bayesian approach. The study concludes that PCR could be used as an effective and rapid test for confirmation of bTB-like lesions detected at slaughtering, supporting current strategies for controlling this disease in endemic countries.

Rift Valley Fever (RFV) is an important zoonotic viral disease of livestock occurring across much of Africa; however, its epidemiology and significance in some parts of the continent are poorly understood. Bronsvoort et al. reported serological evidence of RVF in Central Africa and active circulation of the virus in the cattle population. This study also estimated the performance of a commercial RVF ELISA (ID.Vet) compared with that of a neutralization test (PRNT80), using a Bayesian no gold standard latent class analysis, concluding that the ELISA test had comparable performance and could be used as a low cost easy to use surveillance tool for the African context.

*Salmonella* is an important foodborne pathogen, with pork being one of the major sources of human outbreaks. Surveillance for this pathogen in pigs is important to understand prevalence and distribution. De Lucia et al. investigated the correlation of anti-*Salmonella* antibodies between serum and saliva samples, with results identifying for the first time anti-*Salmonella* antibodies in pigs' oral fluids and suggesting that saliva samples have the potential to be used for the diagnosis of *Salmonella* infection in pig farms.

Leptospirosis is one of the most widespread zoonotic bacterial diseases, and is endemic in subtropical and tropical countries. However, in some countries there is limited knowledge on the epidemiology of the disease. Alinaitwe et al. reported anti-*Leptospira* antibodies among slaughter cattle in Uganda, mainly against the Tarassovi, Sejroe and Australis serogroups, with seroprevalence being higher among older cattle. Their study compared the performance of the standard microscopic agglutination test (MAT) against a lipL32 based real time PCR (qPCR) assay and reported a higher specificity and negative predictive value for the MAT test compared to the qPCR.

## Conclusion

This collection of papers has evidenced the current areas of interest in animal health surveillance developments, from One health to syndromic and risk-based surveillance, from public health to wildlife and livestock health, from the laboratory to epidemiological field and analytical studies. We trust readers will enjoy reading the articles in this Research Topic as much as the Editors enjoyed the process of bringing them to you.

## Author Contributions

MM wrote the draft of the editorial that was then revised and amended by the rest of the editors. All authors were involved in editing manuscripts, approaching reviewers and managing the review process, and in supporting each other with the editorial work.

## Conflict of Interest

The authors declare that the research was conducted in the absence of any commercial or financial relationships that could be construed as a potential conflict of interest.
